# Stone Composition in Patients Who Undergo Renal Stone Surgery: Review of 423 Stone Analyses in Southern Iran

**Published:** 2014-01

**Authors:** Mohammad Mehdi Hosseini, Saeed Shakeri, Farhad Manaheji, Alireza Aminsharifi, Shahrokh Ezatzadegan, Maryam Pakfetrat, Mitra Basiratnia, Mahsa Hosseini

**Affiliations:** 1 Department of Urology, Nephrology-Urology Research Center, Faghihi Hospital, Shiraz University of Medical Sciences, Shiraz, Iran;; 2 Department of Nephrology, Nephrology-Urology Research Center, Faghihi Hospital, Shiraz University of Medical Sciences, Shiraz, Iran

## Dear Editor,

Nephrolithiasis is a common urinary problem with a worldwide estimated prevalence rate of 4–20% and a 5-year recurrence rate of 50%. It accounts for significant expense and morbidity. Recurrent stone disease is a major cause of end-stage renal disease, which may eventually lead to renal transplantation. The prevalence rate of stone disease, in the Middle East region and in Iran, is estimated between 1-20% and 5-7%, respectively.^1^^,^^2^ There are several metabolic disturbances leading to renal stone formation, including hypercalciuria, hyperoxaluria, hyperuricosuria, hypocitraturia, and hypomagnesuria, which also have a definite role in stone composition. Although these metabolic disturbances are responsible for most nephrolithiasis cases, renal stone formation may occur in the absence of any metabolic disturbance. Previous evidence from our region (southern Iran) suggests that low 24-hour urine volume, hypercalciuria, and hyperuricosuria are the most common metabolic abnormalities associated with nephrolithiasis.^3^ As renal stones are the only gross evidence of these disorders, their composition can be used for proposing the pathogenesis leading to stone formation and can provide crucial information for the management of the patients. It has been recommended that the analysis of stone composition should be considered as an integral part of evaluation in those with urinary calculi. This approach would be specifically helpful in determining the cause of stone formation and for planning the prevention strategies.^4^ To the best of our knowledge, there are limited data on renal stone composition in the Iranian population.  We performed a 2-year cross-sectional study on patients with renal stones who were candidats for surgical intervention as a sample of patients with “complex” nephrolithiasis. From March 2009 to March 2011, all patients who underwent surgery for renal calculi were enrolled and their stones were analyzed. The predominant composition of each stone was considered as the basis of classification. Of 423 stones, calcium-oxalate stones were the most common type (67%), followed by uric acid stones (27%). These findings were consistent with previous reports from the Middle East region.^5^^,^^6^ In contrast, the only previous study to have addressed renal stone composition in Iran showed that the most common components were whewellite (81.5%), weddellite (40.7%), apatite (69%), and ammonium acid urate.^7^ The mentioned study was done more than 3 decades ago in Tehran, northern Iran. Compared to our findings, it seems that some factors, including changes in the population’s life style and diet, global warming, and geographic factors, may contribute to the differences in the results.

Although we did not evaluate patients who were managed with medical therapy or shock wave lithotripsy, we recommend stone analysis as a basic and cost-effective method for the evaluation of stone-forming patients. It may help to understand the mechanism of lithogenesis and may help care providers to take preventive measures for these patients.
